# Differentiating Sciatica from Hip Osteoarthritis: Diagnostic Challenges and the Role of the Athena Sign

**DOI:** 10.3390/diagnostics16162515

**Published:** 2026-08-10

**Authors:** Evangelos Sakellariou, Evangelia Argyropoulou, Panagiotis Karampinas, Periklis Pelantis, Ioanna Lianou, Dimitrios Christakos, Ioannis Benetos, Vlachos Ioannis, Angelos Kaspiris, Elias Vasiliadis, John Vlamis, Spyros Pneumaticos

**Affiliations:** 1 Department of Orthopaedic Surgery, KAT General Hospital, National & Kapodistrian University of Athens, 14561 Athens, Greece; vagossak@hotmail.com (E.S.); karapana@yahoo.com (P.K.); pelantisperiklis@gmail.com (P.P.); dimitrioschristakos9@gmail.com (D.C.); ioannisbenetos72@gmail.com (I.B.); eliasvasiliadis@yahoo.gr (E.V.); jvlamis@email.com (J.V.); spirospneumaticos@gmail.com (S.P.); 2 Department of Orthopaedics and Traumatology, University General Hospital of Patras, 26504 Patras, Greece; 3 Department of Orthopaedics and Traumatology, “Agios Andreas” General Hospital of Patras—NHS, 26224 Patras, Greece; jlianou@med.uoa.gr; 4 Orthopaedic Department, Hygeia Hospital, 15123 Athens, Greece; ivlachos@mitera.gr

**Keywords:** sciatica, hip osteoarthritis, hip–spine syndrome, intra-articular injections

## Abstract

**Background/Objectives**: Sciatica and hip osteoarthritis (OA) are common causes of lower extremity pain and functional impairment. Because both conditions may produce overlapping symptoms, distinguishing them can be challenging. Misdiagnosis may lead to inappropriate treatment, delayed intervention and unnecessary procedures. In addition, the frequent coexistence of hip and lumbar degenerative pathology, commonly referred to as hip–spine syndrome, further complicates diagnostic evaluation. This review aims to examine current diagnostic strategies used to differentiate lumbar radiculopathy from hip OA and to introduce the Athena Sign, which is presented as a preliminary, hypothesis-generating clinical observation requiring prospective validation. **Methods**: A narrative review of the literature was performed focusing on the pathophysiology, clinical presentation, differential diagnosis, physical examination and imaging evaluation of sciatica and hip OA. Particular emphasis was placed on clinical examination techniques and diagnostic injections used to identify the primary pain generator. **Results**: Both conditions share overlapping symptom patterns and inflammatory mechanisms, which may obscure the source of pain. Careful assessment of gait, hip range of motion, neurological findings and provocative maneuvers remains essential for accurate diagnosis. Imaging findings should be interpreted in conjunction with clinical examination due to the high prevalence of asymptomatic degenerative changes. The Athena Sign, observed with internal and external rotation of the hips in a prone position with the knee flexed, is associated with anterior intra-articular hip pathology and may provide an additional diagnostic indicator in patients with ambiguous hip–spine presentations. **Conclusions**: A systematic diagnostic approach integrating clinical examination, imaging and targeted diagnostic injections is essential for distinguishing between lumbar radiculopathy and hip OA. Recognition of dynamic clinical findings such as the Athena Sign may further improve diagnostic accuracy and guide appropriate management in patients presenting with overlapping hip and lumbar symptoms.

## 1. Introduction

Lower extremity pain is among the most common complaints encountered in orthopedic and spine practice and frequently originates from either the lumbar spine or the hip joint. Among the most prevalent causes are lumbar radiculopathy (sciatica) and hip osteoarthritis (OA). Sciatica affects approximately 3–5% of the adult population, with the highest incidence observed between ages 40 and 60 years [1]. Hip OA is even more prevalent, affecting up to 10–12% of individuals over the age of 60, with radiographic signs present in almost 20–25% of adults older than 65 years [2]. Both conditions represent major contributors to pain, disability and healthcare utilization in aging populations [1,3]. Sciatica typically results from mechanical compression or chemical irritation of spinal nerve roots, mostly the L4–5 or S1 roots, due to intervertebral disc herniation, spinal stenosis or degenerative changes affecting the lumbar spine [1]. In contrast, hip OA is a degenerative joint disorder, which often develops secondary to mechanical overload and is associated with developmental dysplasia, femoroacetabular impingement, trauma, obesity or age-related degeneration [3]. Despite these clear differences in etiology, the clinical presentations of sciatica and hip OA frequently overlap [4]. Classical descriptions suggest that hip OA manifests as anterior groin pain accompanied by stiffness and limited range of motion, while sciatica typically produces radiating pain following a dermatomal distribution below the knee, often accompanied by neurological symptoms such as numbness, paresthesia or muscle weakness [5,6]. However, in clinical practice, buttock pain caused by posterior capsular irritation in hip OA may be mistakenly interpreted as radicular pain originating from the lumbar spine [7]. The diagnostic challenge is further complicated by the frequent coexistence of hip and lumbar spine disorders, a condition commonly referred to as the hip–spine syndrome [8,9]. Epidemiological studies suggest that 20–30% of patients with hip OA also demonstrate radiographic evidence of lumbar degenerative disease [10,11]. Consequently, determining the primary pain generator and the optimal treatment sequence can be challenging. Misdiagnosis may lead to inappropriate interventions and delayed treatment [12]. Emerging evidence suggests that untreated hip OA may alter spinopelvic biomechanics and adversely affect outcomes following lumbar spinal fusion, underscoring the importance of accurate diagnosis and treatment planning [13,14]. Given these challenges, accurate differentiation between sciatica and hip OA requires a structured and evidence-based diagnostic approach. Certain clinical predictors have been shown to strongly correlate with hip pathology, while dermatomal pain distribution, neurological deficits and positive neural tension tests are more suggestive of lumbar radiculopathy [5,6]. Nevertheless, the sensitivity and specificity of individual diagnostic tests remain limited, emphasizing the need for comprehensive clinical evaluation and careful interpretation of imaging findings.

The aim of this article is to review current evidence regarding the diagnostic differentiation between sciatica and hip OA. Particular emphasis is placed on the identification of key clinical features, highly suggestive examination findings, and novel diagnostic tests, as well as the evolving role of advanced imaging modalities and diagnostic injections in distinguishing these conditions. Furthermore, recent evidence regarding the interaction between hip and lumbar spine pathology, including its implications for treatment decision-making and surgical sequencing, is discussed.

## 2. Materials and Methods

This narrative review was conducted to summarize the current evidence regarding the differentiation of hip osteoarthritis and lumbar radiculopathy (sciatica), with particular emphasis on clinical assessment, imaging evaluation, diagnostic injections, and the emerging concept of the Athena Sign.

A literature search was performed using PubMed and Web of Science databases. The final search was conducted on 31 April 2026. Search terms included combinations of the following keywords: “hip osteoarthritis,” “hip pain,” “lumbar radiculopathy,” “sciatica,” “hip–spine syndrome,” “differential diagnosis,” “physical examination,” “clinical signs,” “diagnostic tests,” “diagnostic injections,” and “imaging.”

Original research articles, systematic reviews, narrative reviews, clinical guidelines, and landmark studies published in English were considered for inclusion. Studies addressing epidemiology, pathophysiology, clinical presentation, physical examination findings, imaging modalities, diagnostic injections, and the interaction between hip and lumbar spine pathology were prioritized. Additional relevant references were identified through manual screening of the bibliographies of selected publications. Studies not available in English, conference abstracts without full-text availability, editorials lacking substantive clinical content, letters to the editor, and studies not directly relevant to the differential diagnosis of hip and lumbar spine pathology were excluded from consideration.

The selection of studies was based on their clinical relevance, methodological quality, and contribution to understanding the diagnostic challenges associated with overlapping hip and lumbar spine disorders. Given the narrative nature of this review, a formal risk-of-bias assessment was not performed.

Particular attention was paid to evidence supporting established diagnostic approaches, while the Athena Sign is presented separately as a preliminary, hypothesis-generating clinical observation derived from clinical experience and included to stimulate future investigation. The limitations inherent to a narrative review, including the potential for selection bias and the absence of quantitative synthesis, should be acknowledged when interpreting the findings presented herein.

## 3. Pathophysiology

Although sciatica arises primarily from pathology affecting the lumbar nerve roots and hip OA originates within the hip joint, both of them involve complex interactions between mechanical stress, inflammatory signaling pathways and nociceptive sensitization. These shared mechanisms contribute substantially to the difficulty clinicians face when attempting to differentiate between spinal and hip sources of lower extremity pain.

### 3.1. Sciatica: Mechanical Compression and Neuroinflammatory Amplification

Sciatica, more precisely termed lumbar radiculopathy, most commonly results from compression or irritation of the L4, L5 or S1 spinal nerve roots. The principal underlying cause is intervertebral disc herniation, which accounts for approximately 85–90% of acute cases, although degenerative spinal stenosis, osteophyte formation, ligamentum flavum hypertrophy and foraminal narrowing may also contribute, particularly in older individuals [1,5,15].

Historically, sciatica was regarded primarily as a mechanical compression neuropathy; however, evidence indicates that compression alone does not fully explain its clinical features. Nerve root compression induces vascular, inflammatory, and neurogenic changes that amplify nociceptive signaling and sustain pain [1]. Mechanical compression impairs venous drainage and cerebrospinal fluid circulation around the affected nerve root, increasing endoneurial pressure and potentially causing localized ischemia [16,17]. Reduced oxygen supply compromises axonal function, while disruption of the blood–nerve barrier facilitates the influx of inflammatory mediators into neural tissue, further contributing to nerve root dysfunction [5].

Disruption of this barrier allows inflammatory mediators to infiltrate the nerve root, converting a purely mechanical injury into a mechanical–inflammatory neuropathy. Degenerating nucleus pulposus tissue releases several potent pro-inflammatory molecules, like phospholipase A2, prostaglandin E2, tumor necrosis factor alpha (TNF-α), interleukin-1β (IL-1β), and interleukin-6 (IL-6). These cytokines directly sensitize nociceptors and enhance pain transmission along affected nerve fibers [18]. Experimental studies have demonstrated that exposure of nerve roots to nucleus pulposus material induces marked TNF-α expression, resulting in nerve inflammation and hyperalgesia. Conversely, inhibition of this cytokine has been shown to significantly reduce radicular pain in animal models [19]. Within hours to days after nerve compression, macrophages, T-lymphocytes, and neutrophils migrate to the affected region and release additional cytokines, reinforcing the inflammatory environment. This immune response perpetuates signaling and contributes to ongoing neural irritation [20].

Persistent nerve root irritation may also lead to central sensitization. Prolonged nociceptive input enhances glutamatergic signaling, while suppressing inhibitory neurotransmitter pathways, resulting in amplified pain perception [21]. Collectively, these mechanisms demonstrate that sciatica represents a multifactorial disorder involving mechanical compression, vascular compromise, inflammatory signaling and central nervous system plasticity.

#### Hip Osteoarthritis: Cartilage Degeneration, Synovial Inflammation and Nociceptor Sensitization

Hip OA is a progressive degenerative joint disorder. Although traditionally described as a mechanical wear-and-tear disease, contemporary research has demonstrated that OA involves a complex interaction between biomechanical stress and inflammatory processes [3]. The pathological process typically begins when mechanical loading exceeds the adaptive capacity of articular cartilage [22]. Mechanical stress activates intracellular signaling pathways within the chondrocytes, particularly mitogen-activated protein kinase (MAPK) and nuclear factor-κB (NF-κB) pathways. These pathways stimulate the production of catabolic enzymes that degrade the cartilage extracellular matrix [23]. Matrix metalloproteinases (MMPs), especially MMP-13, play a critical role in this process by degrading type II collagen, which is the primary structural component of articular cartilage. At the same time, aggrecanases such as ADAMTS-4 and ADAMTS-5 degrade aggrecan, a proteoglycan responsible for maintaining cartilage hydration and compressive resilience [24]. Progressive loss of these structural components weakens the cartilage matrix and leads to thinning, fissuring and eventually loss of articular cartilage. As cartilage deterioration progresses, mechanical forces are increasingly transmitted to the underlying subchondral bone. This altered load distribution stimulates bone remodeling, resulting in subchondral sclerosis, cyst formation and osteophyte development, which represent the hallmark radiographic features of advanced OA [25]. Inflammation of the synovial membrane also plays a key role in OA pathogenesis. Cartilage degradation products act as damage-associated molecular patterns that activate synovial fibroblasts and macrophages. These cells release inflammatory mediators, including IL-1β, TNF-α, and IL-6, which accelerate cartilage degradation while simultaneously sensitizing nociceptive nerve endings within the joint capsule and synovium [2,26].

A notable feature of hip OA is the poor correlation between radiographic severity and pain intensity. This discrepancy is thought to reflect variability in inflammatory activity, nociceptor density and central pain processing mechanisms [2]. The anatomical innervation of the hip joint further contributes to diagnostic complexity. Sensory fibers from the femoral, obturator and sciatic nerves provide overlapping innervation to the joint capsule, allowing hip pathology to produce referred pain in the groin, anterior thigh, buttock and occasionally the knee. In advanced disease, capsular contracture and altered hip biomechanics may increase tension on surrounding neural structures, sometimes producing symptoms that closely resemble lumbar radiculopathy [6,7].

### 3.2. Pathophysiological Convergence and Diagnostic Implications

Despite their distinct anatomical origins, sciatica and hip OA share several important biological features. Both conditions involve the release of inflammatory cytokines, such as TNF-α and IL-1β, which sensitize peripheral nociceptors and contribute to pain amplification. In addition, chronic nociceptive input from either pathology may induce central sensitization, further blurring the clinical distinction between spinal and hip sources of pain [8,26].

This pathophysiological convergence helps explain why patients with either condition may present with pain in similar anatomical regions, particularly the buttock and proximal thigh. A thorough understanding of individual diagnostic tests emphasizes the importance of a systematic approach when evaluating patients with complex hip–spine symptomatology.

### 3.3. Differential Diagnosis

Pain affecting the hip, buttock, thigh or lower extremity might originate from intra-articular hip pathology, periarticular soft structures, the lumbar spine or inflammatory disorders. Because symptom patterns frequently overlap, clinicians must adopt a systematic approach that integrates clinical history, physical examination and targeted imaging in order to identify the primary pain generator [1,27].

#### 3.3.1. Intra-Articular Hip Pathology

Several intra-articular hip disorders may mimic the clinical presentation of lumbar radiculopathy or hip OA. Femoroacetabular impingement (FAI) is among the most common of these conditions. Patients with FAI frequently present with anterior groin pain that may radiate to the thigh, particularly during activities requiring hip flexion and internal rotation [28,29]. Acetabular labral tears represent another important intra-articular cause of hip pain. These injuries may occur secondary to trauma or structural abnormalities such as FAI or degenerative changes associated with early osteoarthritis. Because pain may radiate towards the buttock or thigh, labral tears may occasionally be mistaken for radicular symptoms originating from the lumbar spine [30,31]. Avascular necrosis (AVN) of the femoral head should also be considered, especially in patients with risk factors such as corticosteroid use, alcohol abuse, trauma or systemic disease [32]. Early AVN may present with vague groin or thigh pain and minimal radiographic findings, making magnetic resonance imaging (MRI) essential for early detection [33,34].

Other intra-articular conditions that may resemble hip OA include crystalline arthropathies such as calcium pyrophosphate deposition disease (CPPD) and septic arthritis, particularly in patients presenting with acute onset of symptoms or systemic signs of infection. Failure to recognize infectious etiologies can lead to significant joint destruction and systemic complications if treatment is delayed [35,36,37].

#### 3.3.2. Extra-Articular Hip Pathology

One of the most common causes is greater trochanter pain syndrome, a term encompassing gluteal tendinopathy, trochanteric bursitis and external snapping hip syndrome. Because this pain may radiate toward the lateral thigh, it is sometimes misinterpreted as radicular pain [12].

Iliopsoas bursitis, an internal snapping hip syndrome, represents additional extra-articular sources of hip pain. These conditions typically produce anterior hip discomfort associated with movement or activity and may coexist with intra-articular pathology, further complicating the clinical diagnosis [31,38]. Less common conditions include ischiofemoral impingement, characterized by narrowing of the space between the ischial tuberosity and the lesser trochanter and subspine impingement, which results from contact between the femoral neck and a prominent anterior inferior iliac spine. Both disorders may present with deep buttock or groin pain that mimics lumbar radiculopathy [7,8,39].

#### 3.3.3. Lumbar Spine and Neurological Causes

**Figure 1:**
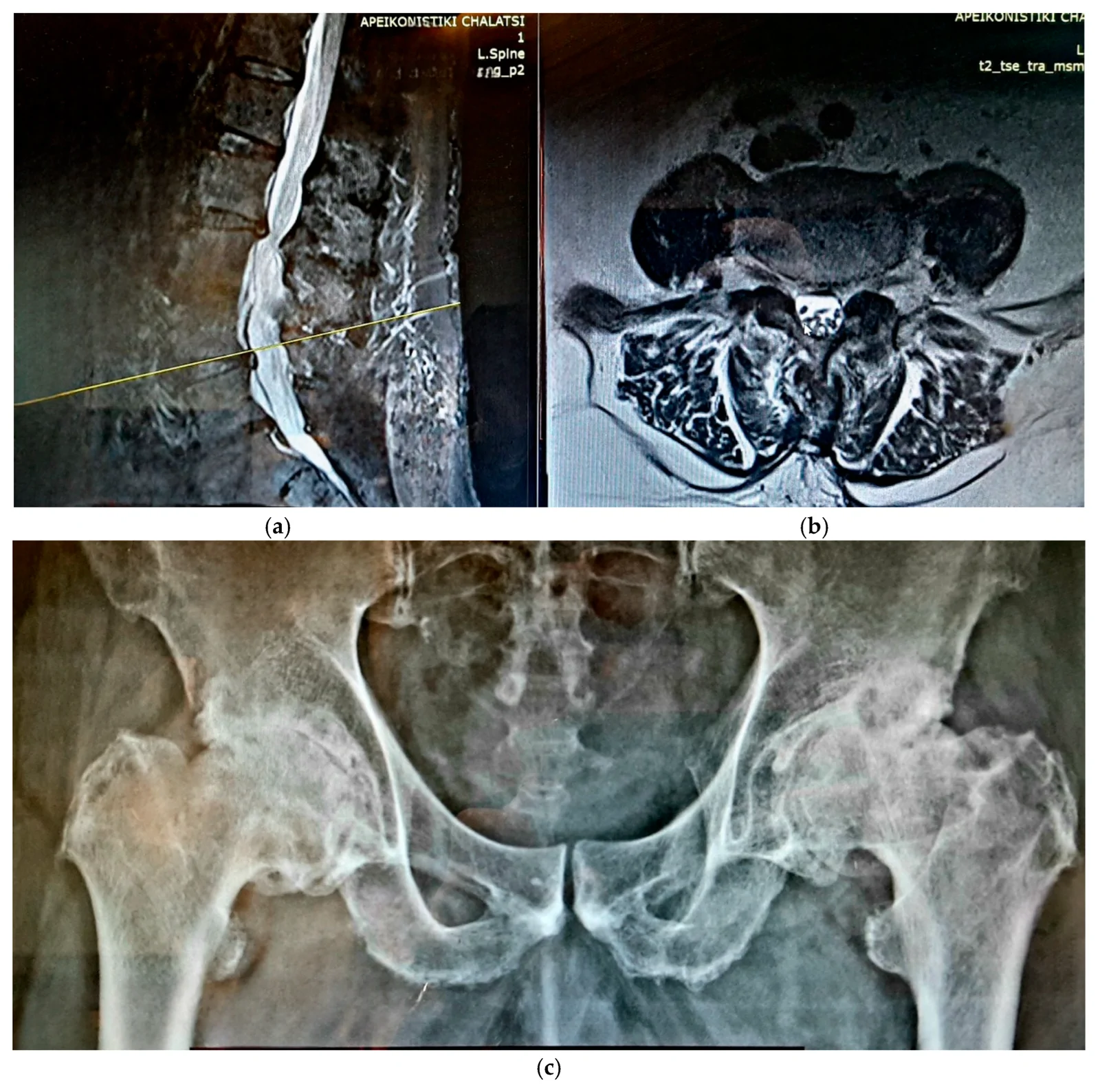
(**a**,**b**) MRI of the lumbar spine showing disc herniation in a patient who was surgically treated for sciatica symptoms. (**c**) X-ray of the hip demonstrating advanced osteoarthritis, which was the actual source of the pain, highlighting the need for differential diagnosis.

Lumbar disc herniation remains the most common cause of sciatica and typically produces dermatomal pain radiating below the knee, often accompanied by neurological deficits such as numbness, paresthesia or muscle weakness [20,40]. Lumbar spinal stenosis may produce neurogenic claudication characterized by leg pain, numbness or weakness that worsens with walking and improves with lumbar flexion. Because symptoms may involve the buttock and proximal thigh, spinal stenosis may occasionally be mistaken for hip pathology [11], as shown in Figure 1.

Other neurological conditions include sacroiliac joint dysfunction, peripheral neuropathies and piriformis syndrome. Although the existence of piriformis syndrome remains debated, it is frequently included in the differential diagnosis of sciatica-like symptoms [9,41,42].

#### 3.3.4. Systemic and Inflammatory Disorders

Systemic diseases may occasionally produce symptoms that mimic either hip or lumbar pathology. Inflammatory arthritis, including rheumatoid arthritis, ankylosing spondylitis and psoriatic arthritis, may affect both the hip and spine, producing pain and stiffness that resemble degenerative conditions [23,43]. Similarly, metabolic and infectious conditions, including tuberculosis, brucellosis and other forms of septic arthritis, may involve the hip joint and present with symptoms that overlap with osteoarthritis or lumbar radiculopathy.

In addition to degenerative and mechanical causes, inflammatory disorders should be considered in patients presenting with overlapping hip and lumbar symptoms. Rheumatoid arthritis, ankylosing spondylitis, and psoriatic arthritis may involve both the axial and peripheral skeleton, potentially resulting in diagnostic uncertainty. In particular, psoriatic arthritis demonstrates considerable heterogeneity, with patients exhibiting predominantly axial, peripheral, or mixed phenotypes that may mimic hip osteoarthritis, lumbar radiculopathy, or hip–spine syndrome. Recent real-world evidence has further highlighted the diverse clinical characteristics, disease burden, and treatment responses observed among patients with axial and peripheral manifestations of psoriatic arthritis, underscoring the importance of accurate phenotypic characterization in routine clinical practice [44,45]. Accordingly, clinicians should maintain a high index of suspicion for inflammatory arthropathies in patients presenting with atypical symptoms, prolonged morning stiffness, elevated inflammatory markers, or a personal or family history of autoimmune disease.

### 3.4. Diagnostic Implication

The extensive overlap between these conditions highlights the importance of a structured diagnostic approach when evaluating patients with lower extremity pain. Clinicians must integrate patient history, perform a physical examination, and perform appropriate imaging studies. Particular attention should be paid to pain localization, radiation patterns and functional limitations. Groin-dominant pain with restricted hip range of motion strongly suggests intra-articular hip pathology, whereas dermatomal pain extending below the knee accompanied by neurological deficits favors lumbar radiculopathy [6,38]. Nevertheless, the coexistence of hip and spinal pathology, commonly referred to as hip–spine syndrome, may further complicate decision-making. In summary, recognition of potential conditions mimicking this condition is critical in order to avoid diagnostic error, prevent inappropriate interventions and guide appropriate treatment strategies for patients presenting with complex musculoskeletal symptoms.

### 3.5. Clinical Presentation and Physical Examination

Although modern imaging modalities have significantly improved diagnostic capabilities, physical examination remains a cornerstone of musculoskeletal assessment and often provides the first clues regarding the anatomical origin of a patient’s symptoms. Pain patterns, gait abnormalities, neurological findings and joint-specific provocative tests can collectively guide clinicians toward the correct diagnosis when interpreted within the broader clinical context [46].

#### 3.5.1. Pain Distribution and Functional Characteristics

Hip OA classically presents with anterior groin pain, which may radiate to the anterior thigh or occasionally to the knee. Patients frequently report stiffness, particularly after periods of inactivity and progressive limitations in activities involving hip flexion or weight-bearing [47]. In contrast, lumbar radiculopathy typically produces radiating pain following a dermatomal distribution, often extending below the knee and accompanied by neurological symptoms such as numbness, paresthesia or muscle weakness [40].

Despite these classical descriptions, symptom patterns frequently overlap in clinical practice. Buttock pain may arise from posterior capsular irritation in hip OA but may also reflect radicular pain originating from lumbar nerve root compression. Similarly, proximal thigh discomfort caused by radiculopathy may be misinterpreted as hip pathology. For this reason, functional limitations often provide additional diagnostic clues [48].

#### 3.5.2. Gait Analysis

Individuals with hip OA frequently demonstrate an antalgic gait, characterized by reduced stance time on the affected limb, in order to minimize pain during weight-bearing. In more advanced disease, weakness or pain inhibition of the hip abductors may produce a Trendelenburg gait, in which the pelvis drops toward the contralateral side during single-leg stance [49,50]. In contrast, patients with lumbar spinal stenosis often adopt a forward-flexed posture while walking, a phenomenon sometimes described as the “shopping cart sign” [11].

#### 3.5.3. Neurological Examination

Motor testing should assess key myotomes, including L4 (knee extension), L5 (ankle dorsiflexion and hip abduction) and S1 (plantarflexion). Sensory testing along dermatomal distributions and assessment of reflexes, particularly the patellar and Achilles reflexes, may reveal abnormalities consistent with nerve root compression [8,51]. However, neurological findings are not always present, particularly in early radiculopathy. Furthermore, pain-related muscle inhibitions may mimic true neurological weakness.

#### 3.5.4. Provocative Hip and Neural Tension Tests

Among the most widely utilized tests are the FABER test (Flexion–Abduction–External Rotation) and the FADIR test (Flexion–Adduction–Internal Rotation), both of which assess intra-articular hip pathology [52,53]. Pain localized to the groin during these maneuvers strongly suggests intra-articular hip pathology. Neural tension tests, including the straight leg raise (SLR) and crossed straight leg raise, are commonly used to evaluate lumbar radiculopathy. However, the specificity of the SLR test is relatively limited, as hip pathology may provoke discomfort during the maneuver [54]. Because individual examination tests often have limited diagnostic accuracy, clinicians typically rely on clusters of findings rather than isolated maneuvers when determining the likely source of symptoms.

### 3.6. The Athena Sign: A Preliminary Clinical Observation

During routine clinical evaluation of patients presenting with anterior hip pain and concomitant or ambiguous lumbar symptoms, one of the senior authors (V.I.) and colleagues observed a reproducible dynamic finding that may assist in raising suspicion for intra-articular hip pathology. The Athena Sign is presented here as a preliminary, hypothesis-generating clinical observation and should not be interpreted as an established diagnostic test.

#### 3.6.1. Technique

The maneuver can be considered a modification of the reverse Thomas test [55,56]. The maneuver is performed with the patient in the prone position with both knees flexed to approximately 90°. The examiner sequentially applies gentle internal and external rotation to the affected hip while maintaining passive hip extension. A positive finding is defined as a reproducible compensatory pelvic elevation occurring during rotational stress of the symptomatic hip. Specifically, internal rotation is associated with elevation of the contralateral hemipelvis, whereas external rotation results in elevation of the ipsilateral hemipelvis. The maneuver may reproduce anterior hip discomfort in patients with suspected intra-articular pathology.

#### 3.6.2. Proposed Biomechanical Basis

The proposed biomechanical basis of the Athena Sign is consistent with established provocative hip examination maneuvers. Hip extension and rotation increase tension across the iliofemoral ligament, anterior capsule, and acetabular labrum, potentially reproducing symptoms in patients with intra-articular pathology. The resulting pelvic compensation may represent a protective neuromuscular mechanism aimed at reducing tension within the symptomatic hip joint. Consequently, the Athena Sign may serve as an adjunctive clinical observation in patients presenting with overlapping hip and lumbar symptoms.

#### 3.6.3. Limitations and Future Directions

Several important limitations should be acknowledged. The Athena Sign has not undergone prospective clinical validation, and no data are currently available regarding its sensitivity, specificity, positive or negative predictive values, or intra- and inter-observer reliability. Furthermore, its performance in patients with mixed pathology, including hip–spine syndrome, remains unknown. Potential examiner-dependent variability and spectrum bias should also be considered.

Future studies should evaluate the diagnostic accuracy, reproducibility, and clinical utility of the Athena Sign in prospective and multicenter cohorts, with comparison against established physical examination maneuvers and imaging-confirmed diagnoses. Until such evidence becomes available, the Athena Sign should be regarded as a preliminary clinical observation intended to generate further investigation rather than as an evidence-based diagnostic test.

### 3.7. Radiological Evaluation and Imaging Strategy

Degenerative changes in the lumbar spine and radiographic evidence of hip OA are both common in older populations and therefore imaging findings alone may not accurately identify the primary pain generator [3,27]. Consequently, imaging should follow a targeted and sequential diagnostic strategy, guided primarily by clinical history and physical examination. This approach reduces the risk of overdiagnosis, prevents unnecessary procedures and improves diagnostic accuracy in patients presenting with overlapping hip and spine symptoms.

#### 3.7.1. Plain Radiography of the Hip

A standard radiographic assessment typically includes an anteroposterior (AP) pelvis view and, when necessary, a frog-leg lateral projection. These views allow evaluation of the joint space width, osteophyte formation, subchondral sclerosis, cyst formation and femoral head deformity, which represent the characteristic structural features of degenerative hip disease [47]. Although widely used in clinical practice and research, the Kellgren–Lawrence classification has notable limitations [57]. Several studies have demonstrated that radiographic severity does not always correlate with symptom intensity or functional limitation [2,47]. Additional radiographic features may provide clues regarding underlying structural abnormalities contributing to hip pathology. For example, deformities in the femoral head–neck junction may suggest femoroacetabular impingement (FAI), while collapse or sclerosis of the femoral head may raise suspicion for avascular necrosis. In such cases, further advanced imaging is typically required to confirm the diagnosis [29].

#### 3.7.2. Magnetic Resonance and Computed Tomography of the Hip

MRI provides superior visualization of soft tissue structures and early joint changes that may not be detectable on plain radiographs. Furthermore, it can identify labral tears, cartilage defects, bone marrow edema, synovitis and early osteonecrosis, making it particularly valuable in patients with persistent symptoms despite normal or equivocal radiographic findings [33]. In the context of differential diagnosis between hip and spinal pathology, MRI can help confirm the presence of intra-articular hip disease when clinical examination findings, like limited internal rotation, positive FABER or FADIR tests, or the presence of the Athena Sign, suggest a hip-based source of pain [50].

Computed tomography (CT) may also be helpful in selected cases, mostly for evaluating complex bony anatomy in femoroacetabular impingement or for preoperative planning prior to hip preservation surgery.

#### 3.7.3. Imaging of the Lumbar Spine

MRI of the lumbar spine is considered the gold standard for evaluating disc herniation, spinal stenosis, nerve root compression and other degenerative spinal conditions [27]. However, numerous studies have demonstrated that lumbar degenerative changes are frequently present in asymptomatic individuals, especially in older populations. In patients presenting with classic radicular symptoms, including dermatomal pain distribution, neurological deficits and positive neural tension tests, MRI can help confirm the level and severity of nerve root compression. Conversely, when imaging findings fail to correlate with the patient’s clinical presentation, clinicians should reconsider alternative diagnoses, including hip pathology.

#### 3.7.4. Radiographic Pitfalls and Diagnostic Challenges

Degenerative changes in both the hip and lumbar spine increase with age and may not necessarily represent the source of a patient’s symptoms. For example, asymptomatic disc protrusions have been identified in up to 30–50% of adults, while radiographic signs of hip OA may be present in individuals without clinical pain [2,27,46]. This phenomenon creates the potential for diagnostic misattribution, in which clinicians attribute symptoms to imaging findings that may not actually represent the primary pain generator. A structured diagnostic process is therefore essential. Imaging should be used to confirm clinical suspicion rather than to establish a diagnosis independently. Careful correlation between imaging findings and clinical examination results, including gait analysis, range of motion and provocative maneuvers, is critical for identifying the true source of pain.

### 3.8. Diagnostic Tests and Injection-Based Localization

When clinical examination and imaging findings fail to clearly identify the primary source of lower extremity pain, additional diagnostic tools may be required to localize the dominant pain generator. In such cases, image-guided diagnostic injections represent one of the most reliable methods for distinguishing between hip-related and spine-related pathology. These procedures allow clinicians to temporarily anesthetize specific anatomical structures and observe whether the patient’s symptoms improve, thereby helping to determine the origin of pain [1,8]. Diagnostic injections are particularly valuable in patients with suspected hip–spine syndrome, a condition in which degenerative changes in the hip joint and lumbar spine coexist and contribute simultaneously to patient symptoms [10].

#### 3.8.1. Intra-Articular Hip Injections

Under fluoroscopic or ultrasound guidance, a local anesthetic, which can be combined with a corticosteroid, is injected directly into the hip joint. The immediate response to this injection provides important diagnostic information. Significant short-term pain relief following intra-articular anesthetic injection strongly suggests that the hip joint is the primary source of symptoms [6].

Ultrasound-guided injections have become increasingly popular due to their high accuracy, absence of ionizing radiation and ability to visualize the surrounding soft tissues. Fluoroscopy remains a reliable alternative, particularly in complex anatomical cases. Studies have demonstrated that properly performed intra-articular injections achieve diagnostic accuracy rates of 85–90% in identifying hip-generated pain [58,59]. In addition to their diagnostic role, intra-articular injections may also provide temporary therapeutic benefit through the anti-inflammatory effects of corticosteroids [60,61].

#### 3.8.2. Selective Nerve Root Blocks

In cases where lumbar radiculopathy is suspected, selective nerve root blocks (SRNBs) may be performed to determine whether a specific nerve root is responsible for the patient’s symptoms. Relief of radicular pain following the injection suggests that the targeted nerve root is the source of the symptoms [62]. Selective nerve root blocks are particularly useful in patients with multilevel degenerative disease of the lumbar spine [48]. By temporarily anesthetizing individual nerve roots, clinicians can identify the specific level responsible for pain and plan surgical or interventional treatment accordingly. Despite their usefulness, SNRBs are not without limitations. False-positive results may occur due to the spread of anesthetic to adjacent nerve roots or placebo effects [27].

### 3.9. Diagnostic Challenges in Hip–Spine Syndrome

The coexistence of hip and spinal pathology presents one of the most complex diagnostic challenges in musculoskeletal medicine. Degenerative changes in both regions are highly prevalent in older adults and imaging findings alone cannot reliably determine the primary source of pain. In such cases, a stepwise diagnostic strategy is often required.

Typically, clinicians begin with a comprehensive clinical examination, including assessment of gait, hip range of motion, neurological findings and provocative maneuvers, such as the FABER, FADIR and straight leg raise tests. Observations such as the Athena Sign, which reflects compensatory pelvic elevation during passive hip extension, may further suggest the presence of intra-articular hip pathology.

When the clinical picture remains unclear, diagnostic injections may provide critical clarification. For example, significant pain relief following intra-articular hip injection strongly implicates the hip joint as the primary pain generator, whereas relief after SNRB suggests the spine is the origin of symptoms (Table 1).

## 4. Discussion

Differentiating between lumbar radiculopathy and hip osteoarthritis (OA) remains one of the most challenging diagnostic tasks in musculoskeletal medicine. Despite their distinct anatomical origins, both conditions frequently produce pain in overlapping anatomical regions, particularly the hip, buttock and proximal thigh. The present review highlights the importance of integrating pathophysiological understanding, clinical examination, imaging strategies and targeted diagnostic procedures in order to accurately identify the primary pain generator.

A major contributor to diagnostic difficulty is the significant clinical overlap between hip and spinal disorders. Hip OA typically presents with groin pain and restricted range of motion, while lumbar radiculopathy produces dermatomal pain radiating along the lower extremity. However, the classic descriptions frequently fail to capture the variability observed in clinical practice. Hip pathology may produce buttock or lateral thigh pain, while radicular symptoms may manifest primarily in the proximal thigh rather than extending below the knee [4,8].

Compounding this challenge is the high prevalence of degenerative findings detected on imaging in asymptomatic individuals. Lumbar disc protrusions and spinal stenosis are commonly observed on MRI in patients without clinical symptoms, while radiographic signs of hip OA may present in individuals who report minimal pain [2,27]. Consequently, imaging findings alone cannot reliably determine the origin of symptoms and must always be interpreted within the broader clinical context.

The coexistence of degenerative pathology in both anatomical regions has been widely described in the orthopedic literature. Epidemiological studies have demonstrated that a substantial proportion of patients with hip OA also present with lumbar degenerative disease and vice versa [10]. This interaction not only complicates diagnosis but may also influence treatment outcomes, particularly when surgical intervention is considered. Given these complexities, a structured diagnostic framework is essential for evaluating patients presenting with lower extremity pain. Clinical examination remains a critical component of this process. Observations such as gait abnormalities, restricted hip range of motion and neurological deficits may provide valuable diagnostic clues when interpreted collectively rather than individually.

Traditional provocative maneuvers, including FABER, FADIR and SLR tests, are widely used in clinical practice. However, the diagnostic performance of these tests varies considerably [52,53]. The SLR test demonstrates high sensitivity for lumbar radiculopathy but relatively low specificity, as hip pathology may also reproduce discomfort during the maneuver [5,54]. Similarly, hip provocation tests may produce pain in patients with lumbar pathology due to referred symptoms. In this context, the Athena Sign, a preliminary, hypothesis-generating clinical observation, may provide an additional diagnostic clue when evaluating patients with overlapping hip and lumbar symptoms. The sign is characterized by a compensatory posterior pelvic elevation that occurs when passive hip extension causes pain in patients with intra-articular hip pathology. Biomechanically, hip extension with the knee flexed increases tension on the anterior capsule, iliofemoral ligament and acetabular labrum. When these structures are inflamed or structurally compromised, the body may reflexively elevate the pelvis in order to reduce capsular tension. This protective response appears to be absent in patients whose symptoms originate primarily from lumbar or sacroiliac pathology. The Athena Sign therefore represents a preliminary descriptive clinical sign requiring validation. Additional clinical studies are required to quantify its diagnostic sensitivity and specificity; preliminary clinical observations suggest that it may be particularly useful in patients with suspected hip–spine syndrome [63].

When clinical examination and imaging findings remain inconclusive, image-guided diagnostic injections can provide valuable information regarding the source of symptoms. Intra-articular hip injections using local anesthetic have been shown to reliably confirm the hip joint as the primary pain generator when significant immediate pain relief is achieved following the procedure [60,61]. Similarly, SNRBs may help identify the specific spinal level responsible for radicular symptoms in patients with multilevel lumbar degeneration. By temporarily anesthetizing individual nerve roots, clinicians can correlate symptom relief with the anatomical source of nerve compression [1,18]. False-positive results may occur due to anesthetic spread or placebo effects and therefore the results of diagnostic injections should always be interpreted alongside clinical findings and imaging modalities [64].

An additional consideration in patients with coexisting hip and lumbar pathology is the optimal sequencing of treatment. Emerging evidence suggests that addressing hip pathology first in patients with significant hip OA may improve outcomes when lumbar surgery is also required [65]. Untreated hip dysfunction can alter spinopelvic mechanics, increasing mechanical stress on the lumbar spine and potentially contributing to poorer postoperative outcomes following spinal fusion [10,13]. Conversely, severe neurological deficits caused by lumbar nerve root compression may necessitate prioritizing spinal intervention. Consequently, treatment decisions should be individualized and based on careful evaluation of the dominant pathology.

### 4.1. Evolving Diagnostic and Therapeutic Perspectives

The recent literature suggests that the diagnostic spectrum of lumbar radiculopathy is broader than traditionally assumed and frequently extends beyond purely spinal degenerative disease. Patriat et al. reported that coronal STIR MRI sequences improve the detection of extra-spinal abnormalities, including hip and periarticular soft-tissue pathology that may mimic or contribute to radicular pain [66]. Although earlier studies reported lower yields due to more limited imaging coverage, large-field coronal STIR protocols appear to increase diagnostic sensitivity in routine practice. However, these benefits must be balanced against the risk of incidental findings and potential overdiagnosis, highlighting the need for careful clinicoradiological correlation.

In parallel, recent evidence on hip–spine syndrome [67] shows that total hip arthroplasty (THA), particularly bilateral procedures, is associated with a reduced risk of subsequent lumbar decompression and fusion, likely through restoration of spinopelvic biomechanics. These findings suggest that THA may not only alleviate hip-related symptoms but also delay or modify the progression of degenerative lumbar disease in selected patients. Collectively, the current literature supports a multidisciplinary and individualized approach to hip–spine syndrome, integrating detailed spinal assessment, biomechanical considerations, and systematic risk factors to optimize long-term outcomes.

### 4.2. Limitations and Future Directions

Several important limitations of this review should be acknowledged. First, although the Athena Sign has been observed consistently in clinical practice, it has not undergone prospective clinical validation. Consequently, no data are currently available regarding its sensitivity, specificity, positive and negative predictive values, or diagnostic accuracy. Furthermore, both intra- and inter-observer reliability have not been assessed. The performance of the Athena Sign in patients with mixed pathology, including hip–spine syndrome, remains uncertain. Given the frequent coexistence of hip and lumbar disorders, spectrum bias and examiner-dependent variability may influence its clinical applicability. Therefore, the Athena Sign should be interpreted as a preliminary, hypothesis-generating clinical observation rather than an evidence-based diagnostic test.

This manuscript represents a narrative review and is therefore subject to limitations inherent to this methodology, including potential selection bias, the absence of formal risk-of-bias assessment, and a lack of quantitative synthesis. Future investigations should focus on prospective diagnostic accuracy studies, multicenter validation cohorts, and assessments of intra- and inter-observer reliability. Comparative studies evaluating the Athena Sign against established physical examination maneuvers and imaging-confirmed diagnoses will be essential to determine its potential role within evidence-based diagnostic pathways for differentiating hip osteoarthritis from lumbar radiculopathy.

## 5. Conclusions

Differentiating hip osteoarthritis from lumbar radiculopathy remains a significant clinical challenge, particularly in patients with overlapping symptoms and concomitant hip–spine pathology. A comprehensive diagnostic approach incorporating clinical history, physical examination, imaging studies, and, when appropriate, diagnostic injections is essential for accurate diagnosis and optimal patient management. This narrative review summarizes current evidence regarding established diagnostic strategies and highlights the importance of recognizing the complex interplay between hip and lumbar spine disorders. The Athena Sign is presented as a preliminary, hypothesis-generating clinical observation that may serve as an adjunctive finding during clinical evaluation. However, prospective validation studies are required to determine its diagnostic accuracy, reproducibility, and potential role within evidence-based diagnostic algorithms. Continued investigation into novel diagnostic approaches, combined with rigorous clinical validation, may ultimately improve the timely identification and management of patients presenting with overlapping hip and lumbar spine symptoms.

## Figures and Tables

**Table 1:** Summary of the established clinical, imaging, and procedural findings that may aid in differentiating hip osteoarthritis from lumbar radiculopathy. The Athena Sign is included separately as a preliminary clinical observation and should be interpreted with appropriate caution.

Category	Findings Suggestive of Hip OA	Findings Suggestive of Lumbar Radiculopathy	Confirmatory/Adjunctive Tests
Clinical History	Groin pain, pain with weight-bearing, reduced walking tolerance	Dermatomal pain, paresthesia, pain below the knee	—
Physical Examination	Reduced hip ROM, FABER, FADIR, Stinchfield test	SLR, crossed SLR, neurologic deficits	Athena Sign *
Imaging	Pelvic radiographs, MRI hip	Lumbar MRI	Diagnostic correlation
Diagnostic Procedures	Intra-articular injection	Selective nerve root block	Response to injection
Associated Syndromes	Hip–spine syndrome	Hip–spine syndrome	Multidisciplinary assessment

* Athena Sign: Preliminary, hypothesis-generating clinical observation. Prospective validation studies are required before incorporation into evidence-based diagnostic pathways.

## Data Availability

The original contributions presented in this study are included in the article. Further inquiries can be directed to the corresponding author.
